# Correction: Evaluating the health and health economic impact of the COVID-19 pandemic on delayed cancer care in Belgium: A Markov model study protocol

**DOI:** 10.1371/journal.pone.0295992

**Published:** 2023-12-12

**Authors:** Yasmine Khan, Nick Verhaeghe, Robby De Pauw, Brecht Devleesschauwer, Sylvie Gadeyne, Vanessa Gorasso, Yolande Lievens, Niko Speybroek, Nancy Van Damme, Miet Vandemaele, Laura Van den Borre, Sophie Vandepitte, Katrien Vanthomme, Freija Verdoodt, Delphine De Smedt

The ninth author’s name is spelled incorrectly. The correct name is: Nancy Van Damme.

## References

[1] Khan Y, Verhaeghe N, De Pauw R, Devleesschauwer B, Gadeyne S, Gorasso V, et al. (2023) Evaluating the health and health economic impact of the COVID-19 pandemic on delayed cancer care in Belgium: A Markov model study protocol. PLoS ONE 18(10): e0288777. 10.1371/journal.pone.0288777 37903130 PMC10615261

