# Correction: Evaluating the health and health economic impact of the COVID-19 pandemic on delayed cancer care in Belgium: A Markov model study protocol

**DOI:** 10.1371/journal.pone.0306495

**Published:** 2024-06-27

**Authors:** Yasmine Khan, Nick Verhaeghe, Robby De Pauw, Brecht Devleesschauwer, Sylvie Gadeyne, Vanessa Gorasso, Yolande Lievens, Niko Speybroeck, Nancy Van Damme, Miet Vandemaele, Laura Van den Borre, Sophie Vandepitte, Katrien Vanthomme, Freija Verdoodt, Delphine De Smedt

The eighth author’s name is spelled incorrectly. The correct name is Niko Speybroeck.

## References

[1] Khan Y, Verhaeghe N, De Pauw R, Devleesschauwer B, Gadeyne S, Gorasso V, et al. (2023) Evaluating the health and health economic impact of the COVID-19 pandemic on delayed cancer care in Belgium: A Markov model study protocol. PLoS ONE 18(10): e0288777. doi: 10.1371/journal.pone.0288777 37903130 PMC10615261

