# Supplementary material for: Evaluating the health and health economic impact of the COVID-19 pandemic on delayed cancer care in Belgium: A Markov model study protocol
Source: PLoS One. 2023 Oct 30;18(10):e0288777. doi: 10.1371/journal.pone.0288777 (PMC10615261; doi:10.1371/journal.pone.0288777)
Supplement: S1 Appendix — (DOCX) [file pone.0288777.s001.docx]

**S1 Appendix.** Cancer patient selection for the estimation of the direct costs of cancer


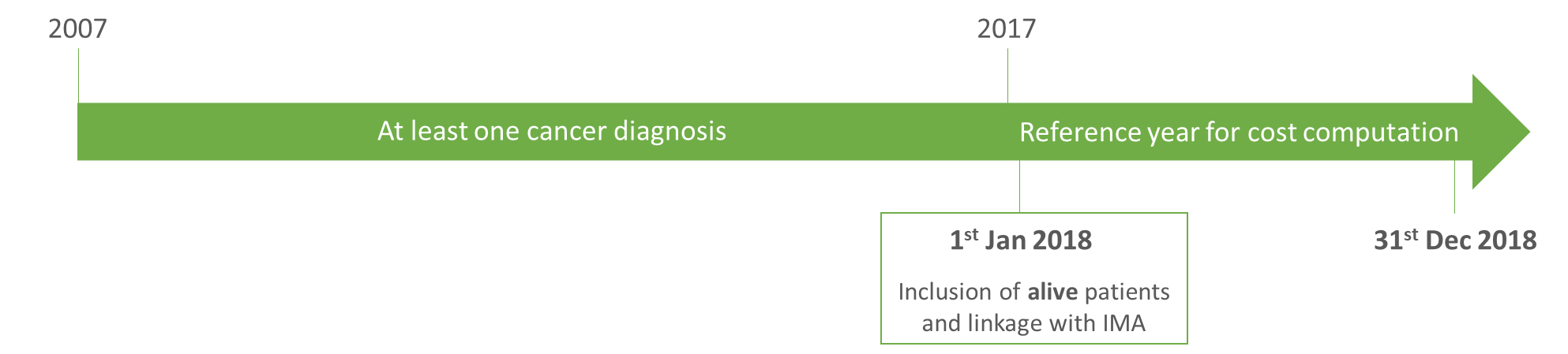
IMA: Intermutualistic Agency
